# A Positioning Method Based on Place Cells and Head-Direction Cells for Inertial/Visual Brain-Inspired Navigation System

**DOI:** 10.3390/s21237988

**Published:** 2021-11-30

**Authors:** Yudi Chen, Zhi Xiong, Jianye Liu, Chuang Yang, Lijun Chao, Yang Peng

**Affiliations:** 1 Navigation Research Center, College of Automation Engineering, Nanjing University of Aeronautics and Astronautics, Nanjing 211106, China; chenyudi@nuaa.edu.cn (Y.C.); ljyac@nuaa.edu.cn (J.L.); yangchuang@nuaa.edu.cn (C.Y.); chaolijun@nuaa.edu.cn (L.C.); 2 Shanghai Aerospace Control Technology Institute, Shanghai 201108, China; 13501798394@163.com

**Keywords:** brain-inspired navigation, place cells, head-direction cells, continuous attractor neural networks (CANNs), population neuron decoding

## Abstract

Mammals rely on vision and self-motion information in nature to distinguish directions and navigate accurately and stably. Inspired by the mammalian brain neurons to represent the spatial environment, the brain-inspired positioning method based on multi-sensors’ input is proposed to solve the problem of accurate navigation in the absence of satellite signals. In the research related to the application of brain-inspired engineering, it is not common to fuse various sensor information to improve positioning accuracy and decode navigation parameters from the encoded information of the brain-inspired model. Therefore, this paper establishes the head-direction cell model and the place cell model with application potential based on continuous attractor neural networks (CANNs) to encode visual and inertial input information, and then decodes the direction and position according to the population neuron firing response. The experimental results confirm that the brain-inspired navigation model integrates a variety of information, outputs more accurate and stable navigation parameters, and generates motion paths. The proposed model promotes the effective development of brain-inspired navigation research.

## 1. Introduction

Unmanned mobile platforms (such as robots, unmanned vehicles, and unmanned aerial vehicles) have a wide range of applications in many industries. For mobile platforms, autonomous navigation is a key technology of automatic operation. At present, the navigation system can be equipped with inertial measurement units (IMU), global navigation satellite systems (GNSS), vision sensors, and radar sensors, etc. However, satellite signals have interfered in satellite-jamming environments (e.g., indoor facilities, tall buildings, forests), which reduces the accuracy of navigation and positioning. Compared with radar sensors and vision sensors, vision sensors have more perceptual information, so the visual autonomous navigation method has been rapidly developed.

In engineering applications, the vision sensor can accurately track environmental features when the mobile platform is moving at a low speed. The use of vision to locate and build maps has achieved good results, but the positioning and navigation effects are not good in the case of weak light and rapid movement of the mobile platform. IMU follows the change of movement speed and accurately measures angular velocity and linear acceleration without the restriction of the scene, but it produces estimated cumulative drift after a long-time operation. In order to take advantage of the respective advantages of vision sensors and IMUs, the fusion of vision and inertial sensor data can provide more accurate position information [1,2]. Location information estimation methods are usually based on probability models, such as extended Kalman filter (EKF) [3], unscented Kalman filter (UKF) [4], and particle filter (PF) [5]. The above methods rely on establishing an accurate navigation system model, which has weak robustness in complex environments. Therefore, a more intelligent way of constructing a reliable navigation model is needed, and here we obtain inspiration from biology in nature.

As we know, mammals such as rats are born with the ability to navigate, and they rely on their conditions to forage, for homing, etc. Animals can locate their position and generate the trajectory of the target position by using self-motion cues, such as vestibule, proprioception, and visual flow [6,7]. It can be seen that the development of brain neuroscience provides a reference and ideas for the development of intelligent autonomous navigation. The neurobiological basis of spatial representation is considered to involve spatially selective neurons in the mammalian nervous system. Some neural basis of navigation has been discovered in the brain, including place cells [8,9], head-direction cells [10], grid cells [11], stripe cells [12], and border cells [13], etc.

With the gradual in-depth study of the brain, the application of brain-inspired navigation technology has attracted more and more attention. Michael et al. proposed a biologically inspired approach to vision-only simultaneous localization and mapping (SLAM), which was called RatSLAM. The system uses the CANN model and visual features to create useful maps of real-world environments [14]. Ball et al. reported an open-source version of RatSLAM, which was bound to the Robot Operating System framework [15]. Steckel et al. suggested changing RatSLAM into BatSLAM by replacing the vision sensor with a biomimetic sonar sensor [16]. Recently, Yu et al. proposed a brain-inspired 4DoF (degrees of freedom) SLAM system named NeuroSLAM based upon computational models of 3D head-direction cells and 3D grid cells, with visual odometry that provides self-motion cues [17]. Zou et al. presented a robotic cognitive map-building approach based on the biological cognitive mechanism of place cells, grid cells, etc. The system is equipped with Kinect vision and a Hokuyo laser sensor, and the input of the neural cell model is speed and direction information [18]. Yuan et al. [19] presented a computational model to build cognitive maps of real environments using both place cells and grid cells, and the RGB-D sensor is used to capture visual images of the environment.

At present, most of the perceptual information input of brain-inspired navigation and positioning methods comes from a single sensor, such as the visual sensor, and lacks the research on decoding navigation parameters from the perceptual information of multiple sensors. Therefore, the positioning accuracy and robustness need to be improved. In this paper, we propose an effective and robust positioning method that combines inertial and visual sensor data for brain-inspired navigation. The creativity of this work is threefold. First, we develop a brain-inspired inertial/visual navigation model for positioning in satellite-jamming environments. Second, we propose a head-direction cell model and place cell encoding model based on continuous attractor neural networks to fuse inertial and visual information. Third, we design a population neuron decoding method to calculate location and direction.

The paper is organized as follows. The second section introduces the brain-inspired navigation model structure and visual information processing. The third section shows the characteristics of the attractor neural network, the encoding method of head-direction cells, and place cells. The decoding method of navigation parameters based on the population of head-direction cells and place cells is explained in the fourth section. The fifth section shows the performance of the proposed model with simulated data and real-world data, experimentally. Finally, the discussion and conclusions are presented separately in the sixth and seventh sections.

## 2. Brain-Inspired Navigation Model

### 2.1. Brain-Inspired Navigation Model Composition

**Figure 1:**
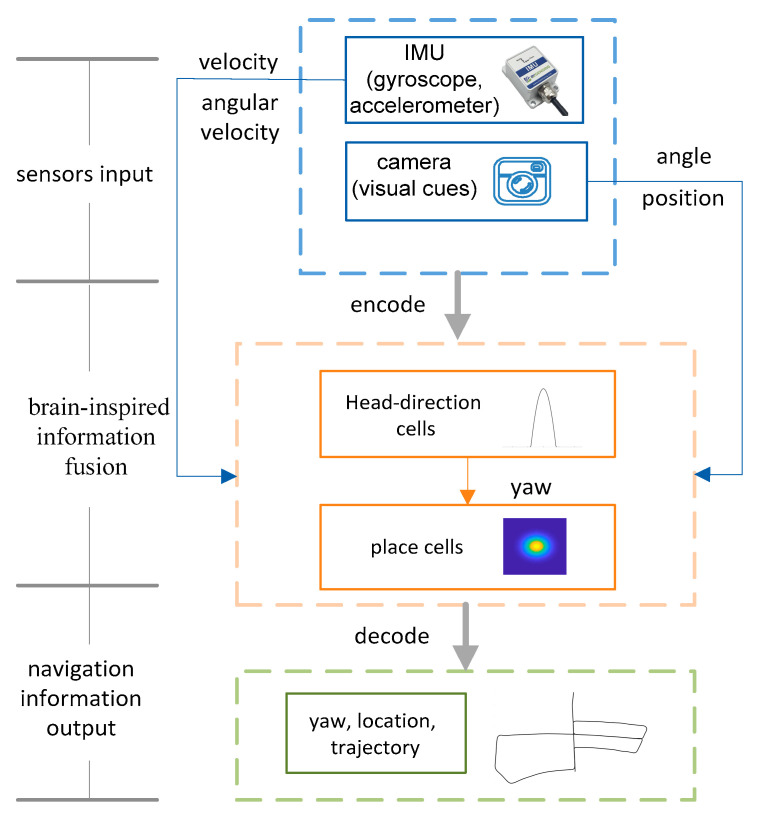
The structure of the brain-inspired positioning model.

The brain-inspired navigation model comprises three main parts: the sensory input module, the brain-inspired information fusion module, and the navigation information output module. Among these modules, the sensory input module consists of an inertial measurement unit (IMU) and a camera, and the inertial measurement unit is composed of a gyroscope and an accelerometer. The brain-inspired information fusion module includes a head-direction cell model and a place cell model. The head-direction cell model fuses the angular velocity provided by the IMU and the yaw angle provided by the visual odometry to obtain the heading, and the place cell model fuses the horizontal velocity of the body coordinate frame from IMU and position provided by visual odometry relative to the initial point. Figure 1 is the structure of the brain-inspired positioning model.

### 2.2. Vision-Based Motion Estimation

The motion estimation is divided into two parts. Firstly, the rotation is estimated, and secondly, the resulting rotation is used to estimate the translation. The visual cues are the grayscale images obtained from the grayscale video camera and the motion state information is obtained through image processing. Image processing includes feature points’ extraction, feature matching, feature selection, and motion estimation. The rotation is estimated by the calibrated monocular camera (left camera), location information is estimated from binocular camera image information, the epipolar constraint between the two images is given as ${q^{\prime }}^{T}Eq=0$, where $q^{\prime }$ and q represent the homogeneous coordinates corresponding to the two images respectively, and E=[t]×R is the 3×3 essential matrix.

The five-point method is used in conjunction with random sample consensus (RANSAC). Several five-point subsets are randomly selected from the total point set, the essential matrix is calculated for each subset, and the essential matrix with the largest interior-point set of all points is selected as the final solution. Equations (1) and (2) impose additional constraints on the five equations [20]:

(1) det(E)=0

(2) $$EE^{T}E-\frac{1}{2}trace(EE^{T})E=0$$

The position information is calculated by binocular vision. After determining all the correspondence between the features, two three-dimensional point clouds can be reconstructed, which are the previous image and the current images respectively, then the position information is calculated by minimizing the image reprojection error. Position information is calculated by iteratively minimizing according to Equation (3):

(3) $$\sum_{i=1}^{n}{\left\|x_{i}^{l}-\pi^{l}(X;R,t)\right\|}^{2}+{\left\|x_{i}^{r}-\pi^{r}(X;R,t)\right\|}^{2}\;\left[\begin{array}{c} u\\ v\\ 1 \end{array}\right]=\pi (X;R,t)=\left[\begin{array}{ccc} f & 0 & c_{u}\\ 0 & f & c_{v}\\ 0 & 0 & 0 \end{array}\right]\left[R|t\right]\left[\begin{array}{c} X\\ Y\\ Z\\ 1 \end{array}\right]\;R|t=\left[\begin{array}{cccc} R_{11} & R_{12} & R_{13} & X\\ R_{21} & R_{22} & R_{23} & Y\\ R_{31} & R_{32} & R_{33} & Z \end{array}\right]$$

where ${\left[\begin{array}{ccc} u & v & 1 \end{array}\right]}^{T}$ are homogeneous image coordinates, π is reprojection function, *t* is the position of the world expressed in the camera coordinate system, and *R* is the rotation matrix which denotes the conversion of the world coordinate system to the camera coordinate system. f is the focal length, ${\left[\begin{array}{cccc} X & Y & Z & 1 \end{array}\right]}^{T}$ are homogeneous world coordinates, which represent the position information calculated by the visual odometry, r represents the right camera and l represents the left camera, and the current rotation is calculated as:

(4) $$\psi^{V}=\arctan(R_{31},R_{33})$$

## 3. Spatial Representation Cells’ Encoding

### 3.1. Continuous Attractor Neural Networks (CANNs)

The model of continuous attractor neural networks (CANNs) as a type dynamics of the neural circuit has been successfully applied to describe the encoding of simple neural systems, such as head-direction, movement direction, and position [21]. The attractor neural network dynamic model has an excitatory recurrent connection and an inhibitory effect between local neurons, so it maintains a stable state by the excitatory recurrent connection and an inhibitory effect without external input. The stable state has the shape of the Gaussian function, which is called ‘bump’. In the steady state, the energy in the CANNs is balanced. The external input breaks the system equilibrium and drives the bump to move. The vertex position of the wave packet is the orientation/position; when the unmanned system moves, the bump in the network follows the movement. For input stimulation close to the preferred direction/position neuron, the overall response of multiple inputs is higher than the single input response, while for input stimulation far away from the preferred orientation/position neuron, the overall response of multiple inputs is lower than the single input response. Based on the characteristics of CANNs and the fusion mechanism, the result of the information calculation is the result of multi-sensors’ interaction [22].

**Figure 2:**
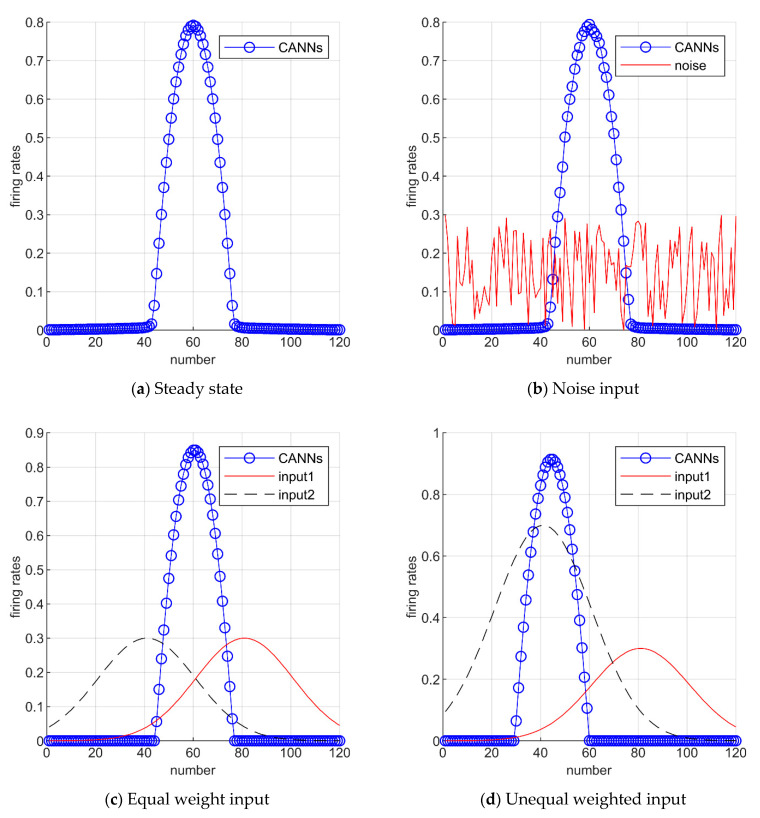
Representation of CANNs properties. (**a**) CANNs are in a stable state. (**b**) The property of the anti-noise. (**c**) When the two input amplitudes of the system are the same, the CANNs model is stable between the two inputs. (**d**) When two inputs have unequal amplitudes, the CANNs model tends to the input with larger amplitudes.

The properties of CANNs in imitating brain neurons’ activities suggested that CANNs serve as a canonical model for information representation [23]. Noise immunity is the important feature of CANNs, and Figure 2 shows the effect of adding noise to the system in CANNs, and the activity is stable. Under the influence of noise, the network model still outputs stable bumps, as shown in Figure 2b. For multiple input sources in the system, another property of the CANNs is the fusion. When the system inputs with two equal-weight signals, the bump center of the CANNs will stabilize between them, otherwise it will tend to the input with larger amplitude, as shown in Figure 2c,d, respectively.

### 3.2. Head-Direction Cells’ Encoding

When mammals face one direction, head-direction cells sensitive to the specific direction (preferred firing direction) will produce the highest firing rate, and the firing morphology is a Gaussian activity package. The cell with the strongest firing rates represents the estimated direction [24]. Therefore, head-direction cells play the role of a “compass” in the brain-inspired system to provide direction.

**Figure 3:**
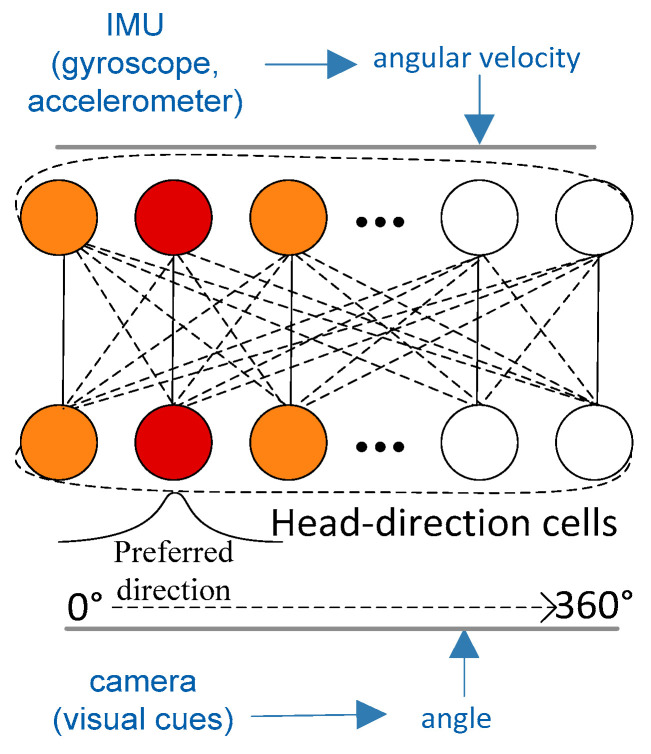
The network architecture of the head-direction cell model. By integrating angular velocity and angle information, the head-direction cell that represents the current direction is activated to generate the maximum discharge (red shades).

In terms of head-direction cell model research, the attractor neural network is often used to construct head-direction cell models [25]. Stringer et al. proposed a model of self-organizing continuous attractor networks for head-direction cells by encoding idiothetic (self-motion) inputs. The model associates the firing with the current orientation change in the representation of the head direction [26]. Xie et al. devised a head-direction system with two populations of neurons which is organized into a ring network structure to mimic head-direction cells, and the system was proven to integrate a large range of the vestibular input [27]. We built a visual and inertial information integration architecture based on the head-direction cell model, as shown in Figure 3. In the figure, each head-direction cell is preset with a preferred firing direction, and these preferred firing directions are evenly distributed to 0°~360°. Head-direction cells integrate the angle calculated by the visual cues and the angular velocity calculated by the IMU. In the process of integrating yaw angle information, the real-time update of the system is achieved through changes in cell activity.

The input data frequency of the head-direction cell model is the same: for visual input, the Gaussian function determines the similarity between the yaw angle calculated by the visual odometry and the preferred direction of the head-direction cell, so the visual odometry data is encoded as a one-dimensional Gaussian distribution to participate in the calculation of the continuous attractor neural network model. The angular velocity calculated by IMU is pre-integrated to obtain the yaw angle. The deviation between the yaw angle and the preferred direction of the head direction cell is encoded as a Gaussian function to obtain the learning firing rates and form an input that participates in data fusion.

The activation level, $h_{i}^{HD}$, of head-direction cell *i* is given by:

(5) $$\tau^{HD}\frac{dh_{i}^{HD}(t)}{dt}=-h_{i}^{HD}(t)+\frac{\phi_{0,\mathrm{HD}}}{C^{HD}}\sum_{j}\left(w_{ij}^{HD}(t)-w^{INH,HD})r_{j}^{HD}(t\right)+I^{V,\psi }(t)+I^{IMU,\psi }(t)$$

where $h_{i}^{HD}(t)$ is the activation level of head-direction cell *i* at time *t*, and $\tau^{HD}$ is the time constant. Head-direction cells are connected by weights, $w_{ij}^{HD}(t)$, which is a function (e.g., Gaussian), $w^{INH,HD}=0.5\times \max(w_{ij}^{HD}(t))$ is a value describing the effect of inhibitory neuron weight, $r_{j}^{HD}(t)$ is the firing rate of head-direction cell *j*, $\phi_{0,\mathrm{HD}}$ is a constant which controls the strength of the weights of head-direction cells, $C^{HD}$ is the number of the cells, and the term $I^{V,\psi }(t)$ represents visual cues’ input.

The firing rate, $r_{i}^{HD}$, of head-direction cell *i* is calculated as the sigmoid function of the activation level:

(6) $$r_{i}^{HD}(t)=\frac{1}{1+e^{-2\beta^{HD}(h_{i}^{HD}(t)-\alpha^{HD})}}$$

where $\alpha^{HD}$ and $\beta^{HD}$ are the sigmoid threshold and slope, respectively.

The weight update according to the Hebbian-like associative learning rule for connections between neurons is as follows:

(7) $$\frac{dw_{ij}^{HD}}{dt}=k\cdot {\bar{r}}_{ij}^{HD}(t){\bar{r}}_{ij}{}^{HD}(t-\Delta t)$$

where $dw_{ij}^{HD}/dt$ is the change of the weight, the learning firing rate, ${\bar{r}}^{HD}$, of each head-direction cell is set as the Gaussian function, *k* is the learning rate constant, which determines the speed of weight change, and $\sqrt{\sum_{j}{\left(w_{ij}^{HD}(t)\right)}^{2}}=1$ denotes that all synaptic weight vectors are normalized after updating at each timestep.

(8) $${\bar{r}}_{ij}^{HD}(t)=e^{-{\left(s_{ij}^{HD}(t)\right)}^{2}/2{\left(\sigma^{HD}\right)}^{2}}$$

(9) $$s_{ij}^{HD}(t)=MIN(\left|\psi_{i}^{HD}-\psi_{j}{}^{ROT}(t)\right|,360-\left|\psi_{i}^{HD}-\psi_{j}{}^{ROT}(t)\right|)$$

In order to integrate IMU information robustly, the preferred angle, $\psi_{j}{}^{ROT}(t)$, modulated by angular velocity is set. Equations (10) to (12) show the modulation process of angular velocity. $\psi_{i}^{HD}$ indicates the preferred direction of the head-direction cell, $s_{ij}^{HD}$ is the difference between the preferred angle modulated by angular velocity and the preferred head direction of the head-direction cell, and $\sigma^{HD}$ is the standard deviation.

(10) $$\left[\psi_{j}{}^{ROT}(t)]=\max({\bar{r}}^{ROT}(t)\right)$$

(11) $$\begin{array}{l} s_{j}^{ROT}(t)=MIN(\left|\psi_{j}^{ROT}-(\psi^{IMU}(t-1)+\omega^{IMU}\cdot \Delta t)\right|\\ ,360-\left|\psi_{j}^{ROT}-(\psi^{IMU}(t-1)+\omega^{IMU}\cdot \Delta t)\right|) \end{array}$$

(12) $${\bar{r}}^{ROT}(t)=e^{-{\left(s_{j}^{ROT}(t)\right)}^{2}/2{\left(\sigma^{ROT}\right)}^{2}}$$

where $s_{j}^{ROT}$ is the difference between the preferred angle modulated by angular velocity and the yaw angle calculated from angular velocity provided by IMU, ${\bar{r}}^{ROT}$ represents the learning firing rate of the preferred angle modulated by angular velocity, $\sigma^{ROT}$ is the standard deviation, $\omega^{IMU}$ is the angular velocity, and °/s is the unit of measurement.

For visual input, $I^{V,\psi }(t)$, and inertial input, $I^{IMU,\psi }(t)$, the angle obtained by sensor data enters the brain-inspired system for direction information integration, and the angle information is encoded by the Gaussian function, as shown in Equation (13):

(13) $$I^{V,\psi }(t)=\lambda^{V}e^{-{\left(s_{i}^{V,\psi }(t)\right)}^{2}/2{\left(\sigma^{V,\psi }\right)}^{2}}\;I^{IMU,\psi }(t)=\lambda^{IMU}e^{-{\left(s_{i}^{IMU,\psi }(t)\right)}^{2}/2{\left(\sigma^{IMU,\psi }\right)}^{2}}$$

(14) $$s_{i}^{V,\psi }(t)=MIN(\left|\psi_{i}^{HD}-\psi^{V}(t)\right|,360-\left|\psi_{i}^{HD}-\psi^{V}(t)\right|)\;s_{i}^{IMU,\psi }(t)=MIN(\left|\psi_{i}^{HD}-\psi^{IMU}(t)\right|,360-\left|\psi_{i}^{HD}-\psi^{IMU}(t)\right|)$$

where $\psi^{V}(t)$ represents the yaw angle obtained by visual information processing, $s_{i}^{V,\psi }(t)$ is the difference between the preferred direction of the head-direction cell and the yaw angle obtained by visual information processing, and $\lambda^{V}$ is the adjustment constant for visual cues. For IMU data input, $I^{IMU,\psi }$, $\psi^{IMU}(t)$ represents the yaw angle calculated by IMU, $s_{i}^{IMU,\psi }(t)$ is the difference between the preferred direction of the head-direction cell and the yaw angle obtained by IMU, and $\lambda^{IMU}$ indicates the adjustment constant of IMU input.

### 3.3. Place Cells’ Encoding

Place cells with spatial selective activity in the hippocampus encode spatial information during navigation and have been proposed to form the neural basis of spatial cognitive maps [28]. When a mammal is in a specific area, the place cells in the brain will produce discharge [29].

**Figure 4:**
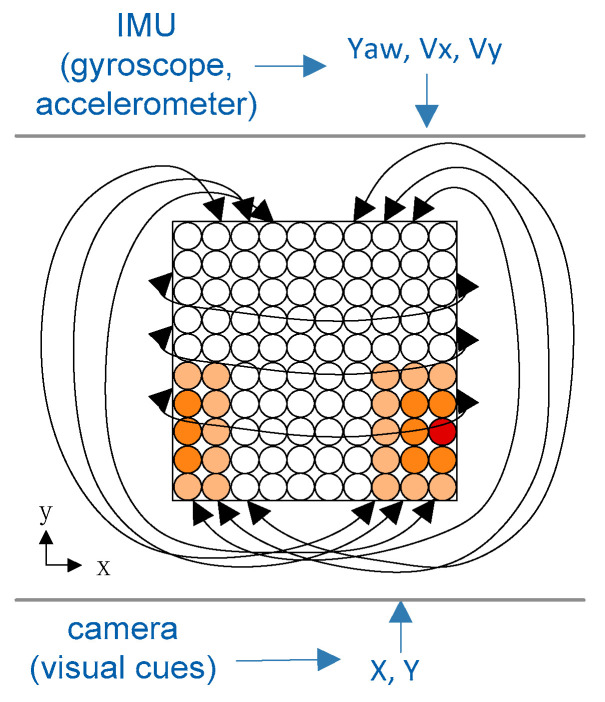
The network architecture of the place cell model. For each neuron, it is activated by the integration of input by IMU and the camera.

The model of place cells draws from the head-direction cell model and can be considered an extension of the one-dimensional CANNs [30]. We present a place cell encoding model for the fusion of visual information and inertial information in Figure 4. The position calculated by the IMU and the position calculated by the visual odometry are both regarded as a two-dimensional normal distribution. Through the interaction, the data from different sensors are exchanged in the continuous attractor neural network model, and the input intensity controls the data intensity involved in the fusion.

The activation level of place cell *i* is given by:

(15) $$\tau^{PC}\frac{dh_{i}^{PC}(t)}{dt}=-h_{i}^{PC}(t)+\frac{\phi_{0,\mathrm{PC}}}{C^{PC}}\sum_{j}\left(w^{PC}(t)-w^{INH,PC})r_{j}^{PC}(t\right)+I^{V,XY}(t)+I^{IMU,XY}(t)$$

where $h_{i}^{PC}(t)$ is the activation level of head-direction cell *i* at time *t*, $\tau^{PC}$ is the time constant for the place cell model, $w^{PC}(t)$ is the excitatory weight, and $w^{INH,HD}$ denotes the inhibitory weight constant. $r_{j}^{PC}(t)$ is the firing rate of the place cell *j*, $\phi_{0,\mathrm{PC}}$ is a constant which controls the strength of the weights of place cells, $C^{PC}$ is the scale of the model, the term $I^{V,XY}(t)$ represents visual cues for positioning, and $I^{IMU,XY}(t)$ denotes the IMU speed input.

The firing rate, $r_{i}^{PC}(t)$, of the place cell *i* at time *t* is calculated as the hyperbolic tangent function of the activation level, and the firing rates of real neurons are greater than zero, thus the firing rate equation is:

(16) $$r_{i}^{PC}(t)=\{\begin{array}{ll} \tanh\left(h_{i}^{PC}(t)\right) & if\;\tanh(h_{i}^{PC}(t))\geq 0\\ 0 & otherwise \end{array}$$

The excitatory weight is created using a two-dimensional Gaussian distribution, as shown in Equation (17):

(17) $$\begin{array}{l} w^{PC}(t)=\frac{1}{\left(\sigma^{PC,X}\sqrt{2\pi }\right)}\cdot \frac{1}{\left(\sigma^{PC,Y}\sqrt{2\pi }\right)}\\ \cdot \exp(-u{\left(t\right)}^{2}/{\left(2\sigma^{PC,X}\right)}^{2})\cdot \exp(-v{\left(t\right)}^{2}/{\left(2\sigma^{PC,Y}\right)}^{2}) \end{array}$$

(18) $$\frac{dw^{PC}}{dt}=k^{PC}\cdot w^{PC}(t)w^{PC}(t-\Delta t)$$

(19) $$w^{PC}(t)=\frac{w^{PC}(t)}{\sqrt{\sum_{i}\sum_{j}w^{PC}(t)}}$$

where $\sigma^{PC,X}$ and $\sigma^{PC,Y}$ are the standard deviation for weight calculation and $k^{PC}$ is the learning rate constant of place cells. Equation (19) denotes that weight vectors are normalized after updating. u(t) and v(t) are the distance indexes between calculated location and the preferred position of the place cells, and the details are as follows:

(20) $$\begin{array}{l} u(t)=\mathrm{rem}(x(t-1)+v_{x}\Delta t-x_{i}^{PC},n_{x})\\ v(t)=\mathrm{rem}(y(t-1)+v_{y}\Delta t-y_{i}^{PC},n_{y}) \end{array}$$

where $n_{x}$ and $n_{y}$ are the half-scale of the place cell model. Adjusting the half-scale parameters of the place cell model is more suitable for engineering applications and corresponding to the navigation coordinate system. It can distinguish and judge the position direction by the positive and negative navigation parameters. u(t) and v(t) are the distance indexes, $x_{i}^{PC}$ and $y_{i}^{PC}$ are the preferred location, and $v_{x}$ and $v_{y}$ are the velocity in the navigation coordinate system, which are calculated by:

(21) $$\begin{array}{l} v_{x}=v_{f}\cdot \sin(\psi )-v_{l}\cos(\psi )\\ v_{y}=v_{f}\cdot \cos(\psi )-v_{l}\sin(\psi ) \end{array}$$

In the above formula, $v_{f}$ and $v_{l}$ are the velocity in the body coordinate system and ψ is the yaw angle.

IMU and visual information are transformed into two-dimensional Gaussian functions, which are expressed as follows, where $I^{IMU,XY}(t)$ indicates IMU information and $I^{V,XY}(t)$ stands for visual information:

(22) $$I^{V,XY}(t)=\lambda^{V,XY}\exp(-{\left(x^{V}-x_{i}^{PC}\right)}^{2}/{\left(2\sigma^{V,X}\right)}^{2}-{\left(y^{V}-y_{i}^{PC}\right)}^{2}/{\left(2\sigma^{V,Y}\right)}^{2})$$

(23) $$I^{IMU,XY}(t)=\lambda^{IMU,XY}\exp(-{\left(x^{IMU}-x_{i}^{PC}\right)}^{2}/{\left(2\sigma^{IMU,X}\right)}^{2}-{\left(y^{IMU}-y_{i}^{PC}\right)}^{2}/{\left(2\sigma^{IMU,Y}\right)}^{2})$$

where $\lambda^{V,XY}$ and $\lambda^{IMU,XY}$ are the adjustment constant, $x^{V}$ and $y^{V}$ represent the two-dimensional position calculated by the visual odometry, $x^{IMU}$ and $y^{IMU}$ represent the two-dimensional position calculated by IMU, and $\sigma^{V,X}$, $\sigma^{V,Y}$, $\sigma^{IMU,X}$, and $\sigma^{IMU,Y}$ are the standard deviation.

## 4. Population Spatial Representation Cells’ Decoding

### 4.1. Population Neuron Decoding

In the previous section, we introduced the spatial representation cell model based on CANNs for motion information encoding, which simulates the discharge response process of a group of neurons receiving external information. Spatial representation cells carry information to support perceptual decisions. In order to use discharge signals for navigation tasks, the brain needs to accurately decode the responses of neurons encoding the perceived information [31].

**Figure 5:**
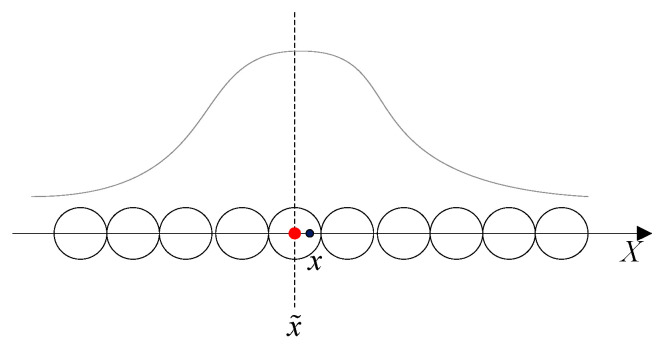
Schematic diagram of population neuron decoding. The red mark indicates the estimated value of the population neuron decoding, and the black mark indicates the true value.

Population neuron decoding is a method to represent neuronal stimulation and obtain accurate information, which can best be used to inform decision-making. A group of neurons form weight distribution according to the different response degree stimulated by perceptual information, so as to estimate the neural response results [32]. Each cell in the population has a preferred direction, and the estimation result is the weighted sum of the preferred directions according to the distribution of the discharge response [33]. As described in Figure 5, visual cues and self-motion information are integrated by the spatial representation cell model to generate discharges for environmental cognition. The center position of the firing activity packet is estimated by the preferred position/direction vector and the firing rates.

### 4.2. Decoding Direction

To decode yaw angle, $\psi_{p}(t)$, from the activity packet formed by the model of head-direction cells’ dynamics, the center of head-direction cells’ firing rates is computed using an established population vector scheme [34]:

(24) $$\begin{array}{cc} \psi_{p}(t) & =\arctan\left(\frac{\sum_{i}r_{i}^{HD}(t)\sin(\psi_{i}^{HD})}{\sum_{i}r_{i}^{HD}(t)\cos(\psi_{i}^{HD})}\right)\\ & =\arctan\left(\frac{a_{pop}}{b_{pop}}\right) \end{array}$$

where $\psi_{i}^{HD}$ is the preferred direction with firing rate $r_{i}^{HD}$.

To make the calculation result meet the range [0, 360°], the above formula is changed to:

(25) $$\psi_{p}(t)=\{\begin{array}{lll} \arctan\left(\frac{a_{pop}}{b_{pop}}\right) & if & a_{pop}>0,b_{pop}>0\\ \arctan\left(\frac{a_{pop}}{b_{pop}}\right)+180^{\circ } & if & b_{pop}<0\\ \arctan\left(\frac{a_{pop}}{b_{pop}}\right)+360^{\circ } & if & a_{pop}<0,b_{pop}>0 \end{array}$$

### 4.3. Decoding Position

The spatial information carried by the firing activity of place cells occupies an important position in animal navigation behavior. Therefore, the place cells’ decoding problem aims to extract information about the location through the firing activity of the cell model [35]. Location information, $x_{p}$ and $y_{p}$, are given as:

(26) $$\begin{array}{l} x_{p}=q^{PC}\cdot n_{x}+\sum_{i}r_{i}^{PC}(t)x_{i}^{pc}\\ y_{p}=q^{PC}\cdot n_{y}+\sum_{i}r_{i}^{PC}(t)y_{i}^{pc} \end{array}$$

where $q^{PC}$ represents the cycle of the real-world location in the framework of the place cell model. $n_{x}$ and $n_{y}$ are the half-scale of the place cell model, $r_{i}^{PC}$ is the firing rate of place cell *i*, and $x_{i}^{pc}$ and $y_{i}^{pc}$ denote the coordinates of the *x*-axis and *y*-axis respectively, in the framework of the position cell model.

## 5. Experiment and Results

### 5.1. Simulation Description

In order to verify the performance of the brain-inspired navigation model, we have carried out a series of experiments. Here, we introduce the experimental content and the experimental environment. We conducted the evaluation on the AMD Ryzen 7 3700X 8-Core CPU 3.60 GHz PC with 8 GB memory windows system. The operating system was Windows 10. Experiments were implemented in MATLAB, which is a powerful and convenient numerical computing platform. The experiments were mainly divided into two parts, namely the simulation data experiment and the real-world data experiment. The simulation data experiment used the trajectory generator to simulate visual data and inertial data, and the KITTI dataset was used in the real-world data experiment. The visual information was obtained by two grayscale cameras, and the parameters were: PointGray Flea2 grayscale cameras (FL2-14S3MC), 1.4 Megapixels, 1/2” Sony ICX267 CCD, global shutter. The inertial information comes from the OXTS RT3003 system, and the sampling rate was 100 Hz.

For our brain-inspired navigation model, Table 1 shows the numerical settings of constant parameters in the head-direction cell model and the place cell model.

### 5.2. Simulation Data Experiment

**Figure 6:**
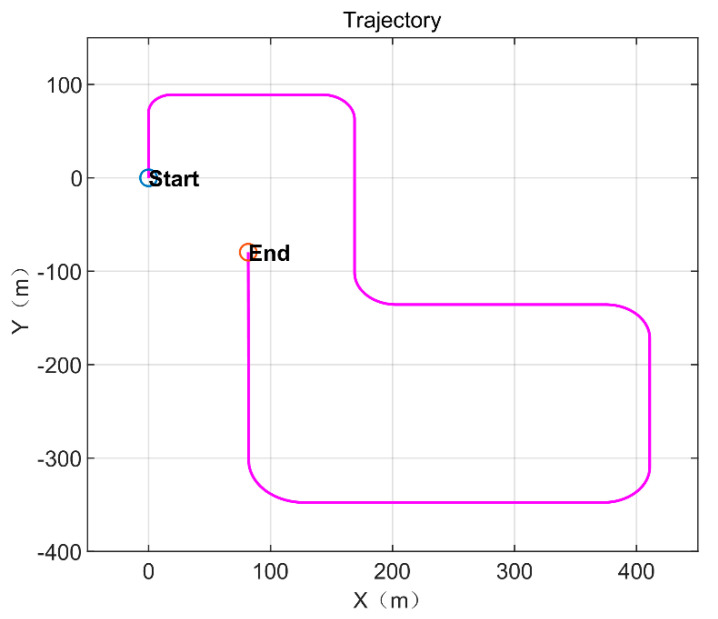
Motion trajectory, the blue circle mark and the red circle mark are divided to indicate the beginning and the end, and the line segment indicates the movement route.

In the simulation data experiment, we designed a plane motion trajectory by simulating the speed and direction of the motion obtained from the IMU, and the position and direction obtained from the visual cues. The true value of the position trajectory is shown in Figure 6, where the navigation coordinate system x and y correspond to east and north, respectively. The whole moving process lasted for 86.1 s, and the data sampling rate was 100 Hz.

**Figure 7:**
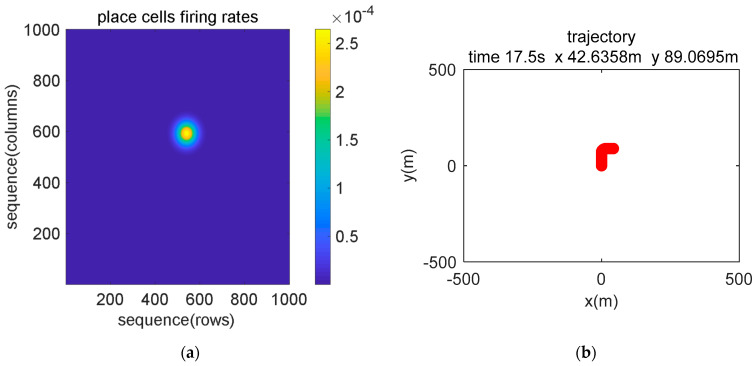

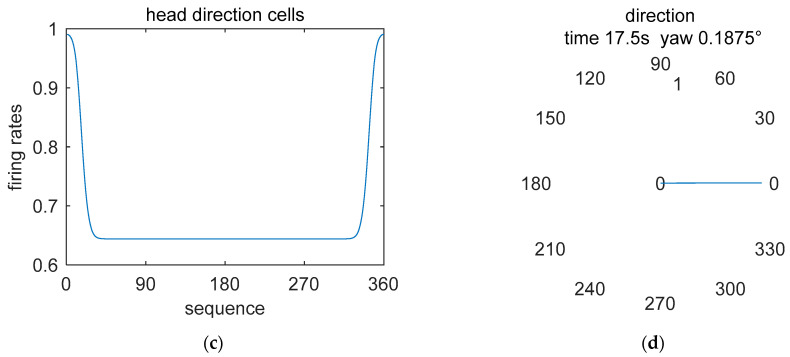
Image display of the simulation data encoding and decoding of the brain-inspired navigation model at the beginning stage. (**a**) Place cell model encodes movement information to generate discharge. (**b**) The location information is decoded and displayed in real-time. (**c**) Head-direction cell model encodes sensor data to simulate discharge. (**d**) The result of yaw angle decoding.

**Figure 8:**
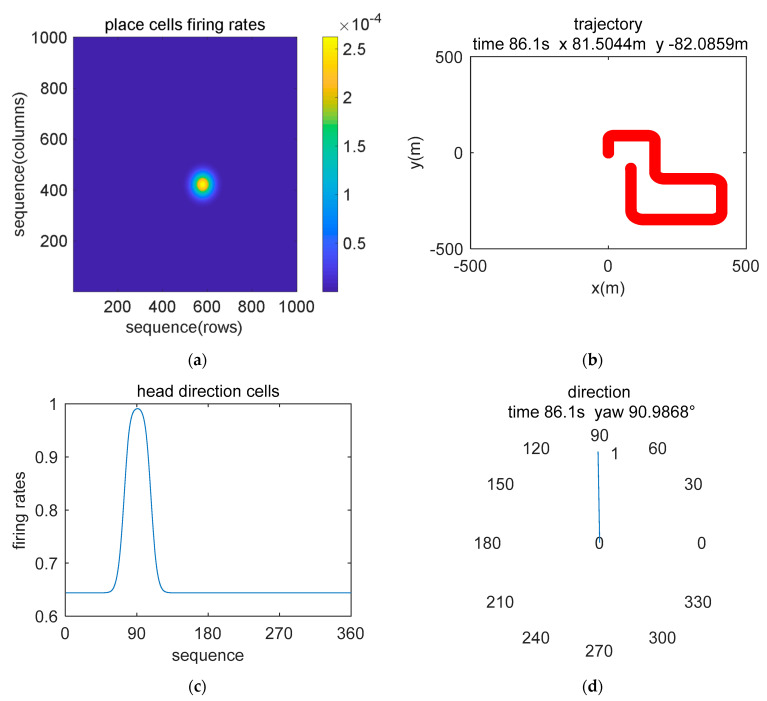
Image display of the simulation data encoding and decoding results of the brain-inspired navigation model at the end of the movement. (**a**) Place cell model encodes movement information to generate discharge. (**b**) The location information is decoded, and the path is displayed. (**c**) The head-direction cell model encodes sensor data to simulate discharge. (**d**) Head orientation at the end of the movement.

Figure 7 and Figure 8 show the experimental results at the beginning and end of the simulation data experiment, respectively. In Figure 7a and Figure 8a, the maximum discharge rate of place cells represents the current position state, and the spatial position was obtained by decoding the discharge rate of the population place cells. The position calculation result is shown in Figure 7b and Figure 8b. In the head-direction cell model, 360 neurons are set to represent 0°~360°, and the model encodes the input data and presents a Gaussian-like distribution, where the largest discharge rate value represents the current direction angle, as shown in Figure 7c and Figure 8c. Figure 7d and Figure 8d show the results of calculating the yaw angle by combining all the head-direction cells and the firing rate. Comparing the results of the current position in the place cell model coordinate system and the navigation coordinate system, it can be verified that our model has the function of simulating the encoding and decoding of spatial representation cells.

**Figure 9:**
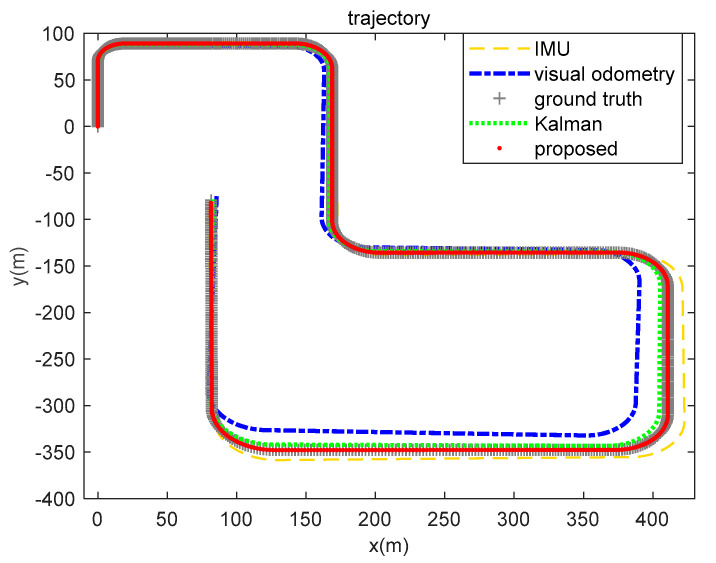
Multiple methods of positioning results and ground truth, including IMU, visual odometry, EKF, and our proposed model.

**Figure 10:**
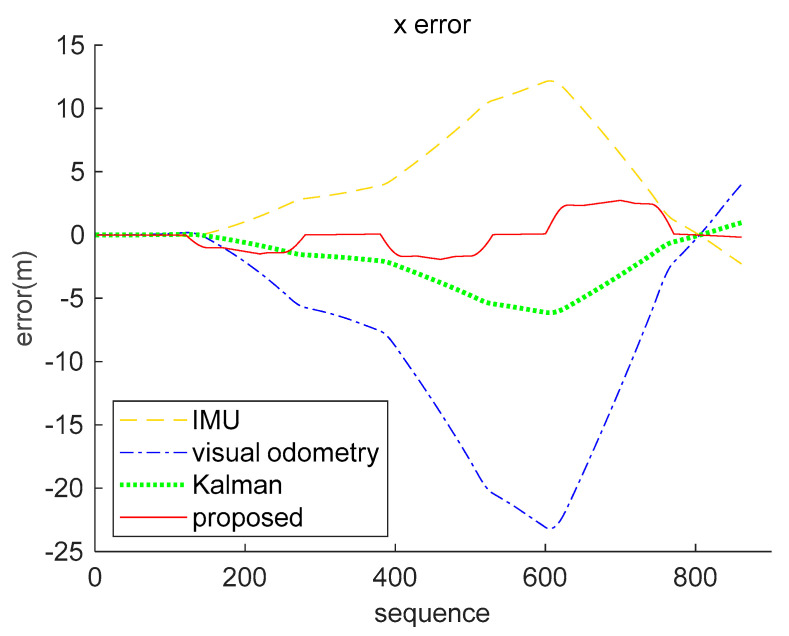
*X*-axis error comparison of simulation data. The yellow loosely dashed line indicates the difference between the *x*-axis position calculated by IMU and the true value, and the blue dash-dotted line represents the difference between the *x*-axis position calculated by the visual odometry and the true value. The green dotted line indicates the *x*-axis position error obtained by the Kalman algorithm fusion of visual and inertial information, and the red solid line represents the *x*-axis position error obtained by our proposed method fusing the visual and inertial information.

**Figure 11:**
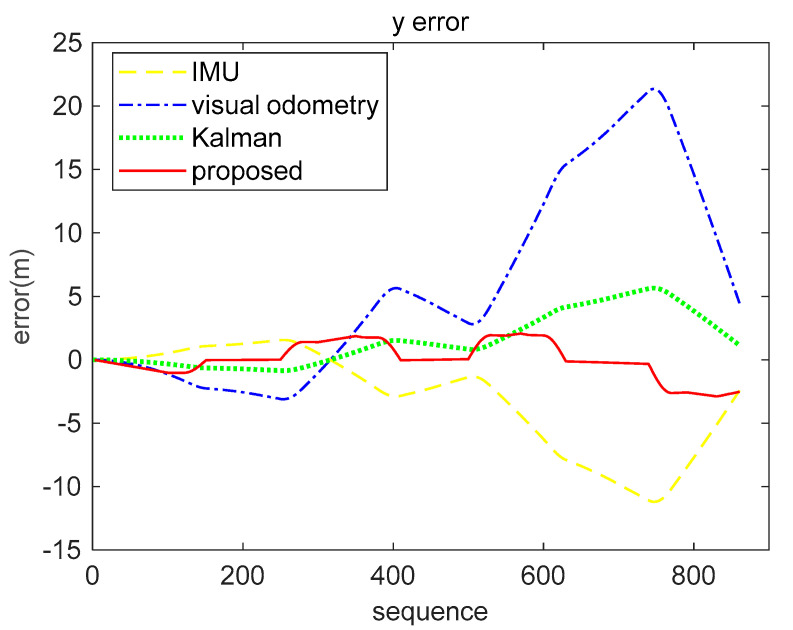
*Y*-axis error comparison of simulation data. The yellow loosely dashed line indicates the difference between the *y*-axis position calculated by IMU and the true value, and the blue dash-dotted line represents the difference between the *y*-axis position calculated by the visual odometry and the true value. The green dotted line indicates the *y*-axis position error obtained by the Kalman algorithm fusion of visual and inertial information, and the red solid line represents the *y*-axis position error obtained by our proposed method fusing the visual and inertial information.

The result analysis of the position data decoded by the brain-inspired navigation model is presented in Figure 9, Figure 10 and Figure 11. Figure 9 is a collection of IMU positioning results, visual odometry positioning results, EKF positioning results, positioning results of our proposed model, and ground truth. In order to distinguish their positioning accuracy more clearly, Figure 10 and Figure 11 show the *x*-axis and *y*-axis errors, respectively. It is not difficult to see from the curve results that both EKF and our proposed model could be more effective in integrating IMU and visual odometry data to achieve the purpose of improving positioning accuracy. However, in the simulation data experiment, the algorithm we proposed performed better in positioning accuracy and information fusion than the algorithm represented by EKF, which requires accurate modeling.

To obtain the positioning evaluation result in numerical form, the root mean square error (RMSE) is used to express the relationship between the estimated position and the true position. Table 2 shows the errors calculated on the x-axes and y-axes by IMU, visual odometry, and our proposed model. The RMSE calculation results show that both EKF and our proposed model can improve the positioning accuracy by fusing the data of IMU and visual odometry. Among them, the data fusion effect of our model is better, and the error between the estimated position and the real data is smaller.

(27) $$\mathrm{RMSE}=\sqrt{\frac{1}{m}\sum_{i=1}^{m}{\left(\Delta e_{i}\right)}^{2}}$$

### 5.3. Real-World Data Experiment

The real-world data come from the KITTI dataset 05 sequence, where pictures collected by the grayscale camera and the raw data of the IMU are used as the system input [36]. The 05 sequence contains 2762 sets of data, the range of motion covers approximately 600 × 500 m, and the path length is about 2117.3 m.

**Figure 12:**
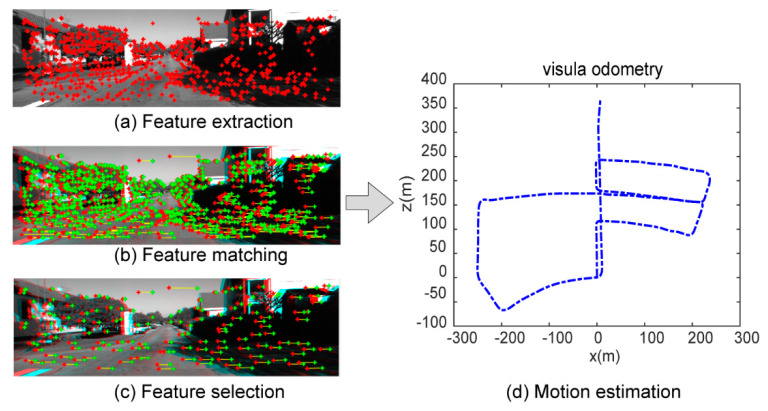
Image processing. (**a**) Feature extraction, the extracted feature points in the grayscale image are marked in red. (**b**) Feature matching, the red marks are feature points in the left image, green marks are features in the right image, and the yellow marks indicate feature matching. (**c**) Feature selection, the graph shows the results of filtering available features. (**d**) Motion estimation is obtained by visual odometry.

In the visual information processing stage:

(1) Feature extraction: each image is extracted with corner and blob features, as shown in Figure 12a.
(2) Feature matching: Starting from all the feature points in the left image at time t, the best matching point is found in the left image at time t-1, and then the feature points are still found in the right image at time t-1 and the right image at time t. The best match is found in four images acquired at consecutive moments, as shown in Figure 12b.
(3) Feature selection: in order to ensure that the features are evenly distributed in the entire image, the entire image is divided into buckets with a size of 50 × 50 pixels, and feature selection is performed to select only the strongest features present in each bucket, as shown in Figure 12c.
(4) Motion estimation: the position information as shown in Figure 12d is estimated by using the perspective three-point (P3P) algorithm and RANSAC [37].

In order to facilitate calculation, the left grayscale camera was selected as the body coordinate system: x corresponds to right, y corresponds to down, and z corresponds to forward. The first frame was used as a reference point to calculate the subsequent relative position coordinates. The sampling frequency of IMU and image data was consistent, which was 100 Hz.

**Figure 13:**
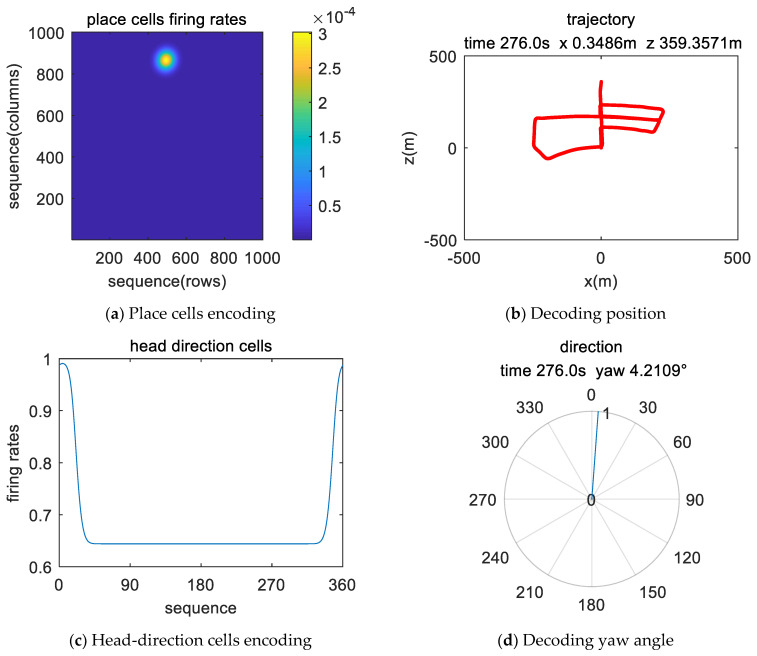
Image display of the real-world data encoding and decoding of the brain-inspired navigation model at the beginning stage. (**a**) Place cell model encodes movement information to generate discharge. (**b**) Decoding location information. (**c**) The head-direction cell model encodes sensor data to simulate discharge. (**d**) Decoding direction information.

The encoding results of image and IMU data by place cells and head-direction cells are shown in Figure 13a,c. Position parameters and yaw angle are shown in Figure 13b,d. From the start to the end, the trajectory lasted for 276 s and covered an area of 500 × 500 m. The maximum discharge position of the place cell changes with the movement position, and the maximum discharge position of the head-direction cell changes with the yaw angle in the range of 0 to 360 degrees.

**Figure 14:**
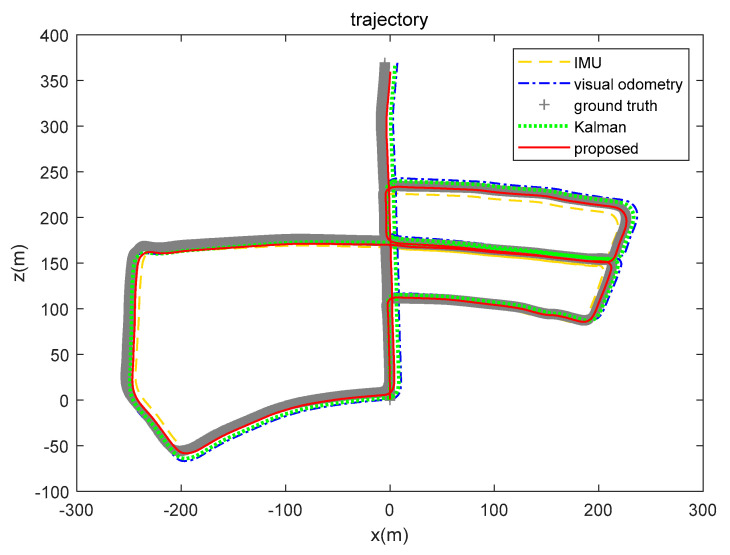
IMU, visual odometry, ground truth, EKF, and brain-inspired model positioning (KITTI dataset 05 sequence). The yellow loosely dashed line represents the trajectory calculated by the IMU, the blue dash-dotted line represents the visual odometry calculation result, the gray line segment represents the ground truth, the green dotted line represents the calculation result of Kalman algorithm fusion of visual and inertial information, and the red solid line represents the positioning result of our proposed algorithm.

In order to analyze the fusion ability and positioning results of the proposed algorithm for real-world environment data, we gathered the trajectory routes of IMU, visual odometry, ground truth, EKF, and the brain-inspired navigation model, as shown in Figure 14. From the perspective of the positioning route, the algorithm we proposed is close to the real value.

**Figure 15:**
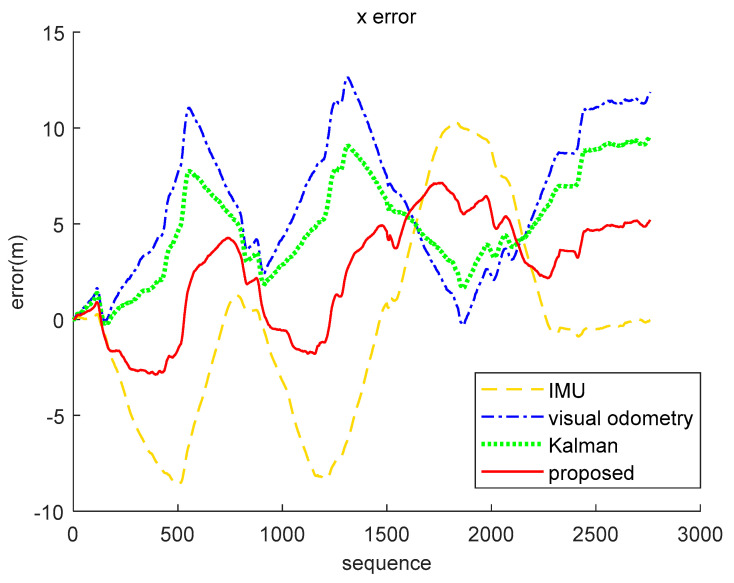
*X*-axis error comparison of real-world data (KITTI dataset 05 sequence). The yellow loosely dashed line indicates the difference between the *x*-axis position calculated by IMU and the true value, the blue dash-dotted line represents the difference between the *x*-axis position calculated by the visual odometry and the true value, the green dotted line indicates the *x*-axis position error obtained by the Kalman algorithm fusion of visual and inertial information, and the red solid line represents the *x*-axis position error obtained by our proposed method fusing the visual and inertial information.

**Figure 16:**
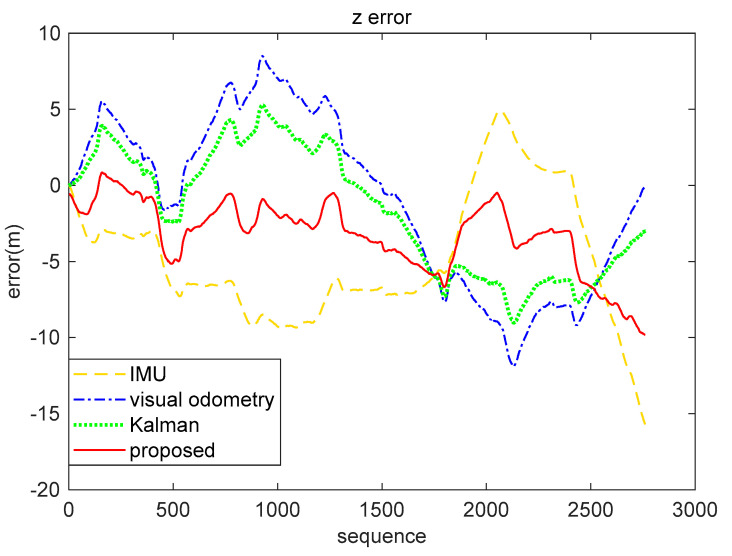
*Z*-axis error comparison of real-world data (KITTI dataset 05 sequence). The yellow loosely dashed line indicates the difference between the *z*-axis position calculated by IMU and the true value, the blue dash-dotted line represents the difference between the *z*-axis position calculated by the visual odometry and the true value, the green dotted line indicates the *z*-axis position error obtained by the Kalman algorithm fusion of visual and inertial information, and the red solid line represents the *z*-axis position error obtained by our proposed method fusing the visual and inertial information.

Respectively, we further analyzed the difference between ground truth and the positioning estimation results of IMU, the positioning estimation results of visual odometry, the positioning estimation results of EKF, and the positioning estimation results of our proposed model. The difference between the positioning estimation result and the true value is drawn into a curve, where the error curves in the *x*-axis and *z*-axis are shown in Figure 15 and Figure 16. It can be seen from the results that the positioning effect of using IMU and visual odometry alone does not work effectively. Using EKF and the model proposed in this paper to fuse data can improve the positioning accuracy. The calculation results of the RMSE in Table 3 illustrate that the brain-inspired navigation model is more effective in terms of data fusion and positioning effects. The *x*-axis direction RMSE was 3.8551 m and the *z*-axis direction RMSE was 3.9532 m, which are less than the calculation results of other methods.

## 6. Discussion

In this paper, we expressed the idea of building a spatial representation model based on attractor neural networks, which provides a new idea for multi-source data fusion. For model input, the direction and position of visual estimation and the angular velocity and velocity collected by IMU were used to participate in the model calculation. However, the essence of the fusion data of the head-direction cell model and the place cell model are angle and position, respectively. It is necessary to unify the frequency of IMU and visual odometry calculation data in advance.

### 6.1. Model Parameter Adjustment

Through experimental verification, the head-direction cell model and place cell model that we proposed integrate visual and IMU data to simulate the discharge morphology of head-direction cells and place cells to encode information. In order to obtain more accurate and reliable orientation and position, we proposed a method to decode information based on the population spatial representation cells. Compared with the EKF algorithm that relies on the probability model, we do not need to spend too much time adjusting the parameters of the brain-inspired model to make data fusion.

As for the parameter adjustment methods in the head-direction cell model and the place cell model, there are two types of parameters that need to be set according to the environment and sensors, respectively: the range of data and the intensity of data fusion. To make the model structure simple and more suitable for engineering applications, the number of head-direction cells, $C^{HD}$, was set to 360, which corresponds to the maximum yaw angle of 360 degrees. The place cell model 1 unit corresponds to 1 m in the real environment. $n_{x}$ and $n_{x}$ are the half-scale of the place cell model. Since the scale of the place cell model is 1 m to the real scale, the half-scale of the place cell model is not less than the maximum absolute value of the position coordinate solved in the navigation coordinate system. $\frac{\phi_{0,\mathrm{HD}}}{C^{HD}}$ and $\frac{\phi_{0,\mathrm{PC}}}{C^{PC}}$ respectively represent a certain ratio to the scale of the head-direction cell model and the place cell model. In the head-direction cell model and the place model, we set them to 4, so we obtained $\phi_{0,\mathrm{HD}}=4\times C^{HD}$ and $\phi_{0,\mathrm{PC}}=4\times C^{PC}$. λ represents the intensity of sensor data input. When the information solution accuracy of IMU and visual odometry is consistent, it is set to 1, otherwise, the weight of the sensor with high-performance navigation parameter solution ability can be increased.

### 6.2. Other Dataset Experiments

**Figure 17:**
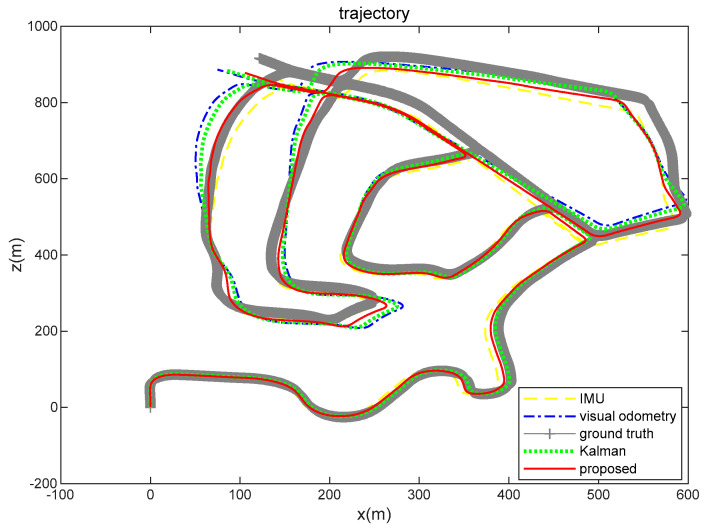
Results of position calculation of KITTI dataset 02 sequence. The yellow loosely dashed line represents the trajectory calculated by the IMU, the blue dash-dotted line represents the visual odometry calculation result, the gray line segment represents the ground truth, the green dotted line represents the calculation result of Kalman algorithm fusion of visual and inertial information, and the red solid line represents the positioning result of our proposed algorithm.

The KITTI dataset 02 sequence was used to verify the model performance in a large-scale environment (the range here was about 600 × 1200 m, and the path length was about 4880 m), and 4661 sets of data were used in the 02 sequence. Due to the expansion of the motion trajectory range, the half-scale of the place cell model was set to 1000, and the other parameters do not follow the environmental adjustment. Figure 17 shows the results of multiple positioning methods, and Table 4 shows the calculation results of the RMSE. According to the pictures and table data, the brain-inspired model we proposed has the ability to fuse the position information of the IMU and the visual odometry, and the location information solution is closer to the real location.

In particular, the positioning effect of the IMU in the *x*-axis direction was better. The RMSE was 8.7314 m, and the visual odometry had a larger error in the *x*-axis direction of 26.7076 m. The fusion algorithm was affected by the large error of the visual odometry. The RMSE of the Kalman algorithm on the *x*-axis was 22.0495 m, and the RMSE of our proposed algorithm was 13.8207 m. The calculation results of the two fusion algorithms in the *y*-axis direction are better than the calculation results using a single sensor. From the comprehensive analysis, experimental data show that our proposed method is slightly better in terms of fusion.

**Figure 18:**
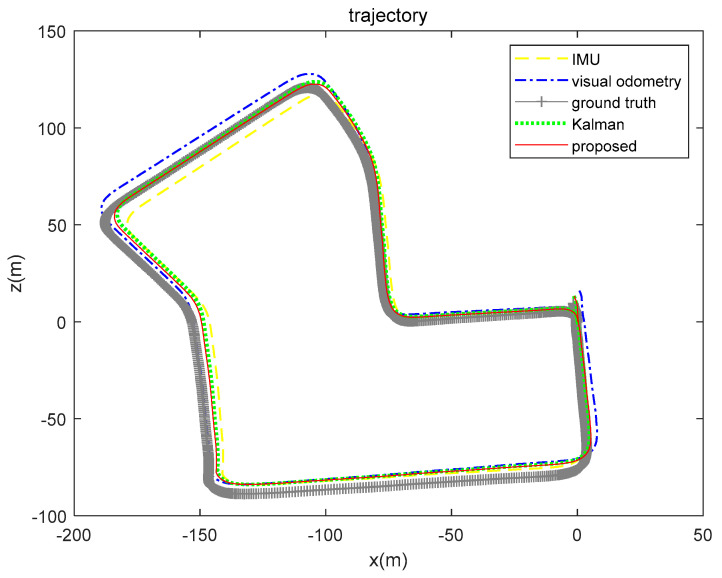
Results of position calculation of KITTI dataset 07 sequence. The yellow loosely dashed line represents the trajectory calculated by the IMU, the blue dash-dotted line represents the visual odometry calculation result, the gray line segment represents the ground truth, the green dotted line represents the calculation result of Kalman algorithm information fusion, and the red solid line represents the positioning result of our proposed algorithm.

The KITTI dataset 07 sequence is a visual closed loop of the movement of the mobile platform. The range is about 600 × 1200 m. Figure 18 shows the motion trajectory estimation results of the 07 sequence. The yellow loosely dashed line and blue dash-dotted line are the trajectories calculated by a single sensor, the yellow loosely dashed line represents the motion trajectory calculated by the IMU, and the blue dash-dotted line is the motion trajectory calculated by the visual odometry. The ground truth shows the actual motion trajectory as a closed loop, however the trajectory of the visual odometry does not form a closed loop. Using the algorithm to fuse the positioning data of the IMU and the visual odometry can reduce the error of only using the visual odometer to form a closed motion loop and make the calculation result closer to the true value. Both the Kalman algorithm and our method have data fusion effects. As can be seen from Table 5, our proposed method integrates IMU and visual odometer data to obtain better positioning results.

In theory, it is especially proposed that the information of brain-inspired model fusion is not limited to vision and IMU, and the position data provided by any sensor can participate in the calculation. The advantage of the brain-inspired positioning model is that the model is constructed inspired by brain neurons and provides some ideas for brain-inspired engineering applications. Our proposed brain-inspired model has a good performance in data fusion, and there is no need to change or rebuild the model according to the different sensors used, so it is suitable for engineering applications. However, this model has some shortcomings when used in a wide range of environments, because the place cell model scale is set in relation to the range of motion. The current experiment was based on the collected dataset and is limited by the computer used for the simulation experiment. The experiment has not been carried out for the calculation delay caused by a large amount of data, so this research can be realized in the future. In general, the number of head-direction cells and place cells is related to the region, and the relationship between the number of cells and the positioning performance needs to be further studied in the future.

## 7. Conclusions

In this paper, we proposed a positioning method based on spatial representation cells for satellite-jamming environments. This method uses attractor neural networks to build the head-direction cell model and the place cell model to encode vision and IMU data, and the navigation parameters are decoded based on the population neurons. Experimental results showed that the proposed model effectively integrates vision and IMU data and provides more accurate position and direction information. In conclusion, our proposed method has the following contributions:

(1) A brain-inspired research framework based on visual and inertial information was provided for the intelligent autonomous navigation system in complex environments.
(2) A brain-inspired visual-inertial information encoding method and navigation parameter methods were proposed to explore brain-inspired research ideas from neuroscience to application.
(3) The brain-inspired navigation model promotes the development of more intelligent navigation systems and provides the possibility for the wide application of brain-inspired intelligent robots and aircraft in the future.

## Figures and Tables

**Table 1:** Parameters’ configuration.

Parameter	Value	Parameter	Value
$C^{HD}$	360	$\phi_{0,\mathrm{HD}}$	1440
$C^{PC}$	1001	$\phi_{0,\mathrm{PC}}$	4004
$\lambda^{V}$,$\lambda^{V,XY}$$,\lambda^{IMU,XY}$	1, 1, 1	$n_{x}$, $n_{y}$	500, 500

**Table 2:** Simulation data experiment, RMSE.

	IMU	Visual Odometry	Kalman (Inertial-Visual)	Proposed (Inertial-Visual)
x(m)	5.8858	11.2778	2.9994	1.2889
y(m)	5.0015	9.6306	2.5736	1.3516

**Table 3:** Real-world data experiment, RMSE.

	IMU	Visual Odometry	Kalman (Inertial-Visual)	Proposed (Inertial-Visual)
x(m)	5.2123	7.1180	5.5708	3.8551
z(m)	6.3941	5.6666	4.3949	3.9532

**Table 4:** KITTI dataset 02 sequence experiment, RMSE.

	IMU	Visual Odometry	Kalman (Inertial-Visual)	Proposed (Inertial-Visual)
x(m)	8.7314	26.7076	22.0495	13.8206
z(m)	24.0802	22.7763	20.4295	19.6386

**Table 5:** KITTI dataset 07 sequence experiment, RMSE.

	IMU	Visual Odometry	Kalman (Inertial-Visual)	Proposed (Inertial-Visual)
x(m)	4.8461	1.8431	3.0742	2.8290
z(m)	2.8955	6.3704	4.5074	3.1663

## Data Availability

Not applicable.
